# PrecivityAD2™ Blood Test: Analytical Validation of an LC-MS/MS Assay for Quantifying Plasma Phospho-tau217 and Non-Phospho-tau217 Peptide Concentrations That Are Used with Plasma Amyloid-β42/40 in a Multianalyte Assay with Algorithmic Analysis for Detecting Brain Amyloid Pathology

**DOI:** 10.3390/diagnostics14161739

**Published:** 2024-08-10

**Authors:** Stephanie M. Eastwood, Matthew R. Meyer, Kristopher M. Kirmess, Traci L. Wente-Roth, Faith Irvin, Mary S. Holubasch, Philip B. Verghese, Tim West, Joel B. Braunstein, Kevin E. Yarasheski, John H. Contois

**Affiliations:** C2N Diagnostics, 4340 Duncan Avenue, Suite 110, Saint Louis, MO 63110, USA; seastwood@c2n.com (S.M.E.); mmeyer@c2n.com (M.R.M.); kkirmess@c2n.com (K.M.K.); twente-roth@c2n.com (T.L.W.-R.); firvin@c2n.com (F.I.); mholubasch@c2n.com (M.S.H.); pverghese@c2n.com (P.B.V.); twest@c2n.com (T.W.); jbraun@c2ndiagnostics.com (J.B.B.); jcontois@c2n.com (J.H.C.)

**Keywords:** Alzheimer’s biomarker, blood biomarker test, diagnostic tool, mass spectrometry, analytical methods/validity, brain amyloid plaques

## Abstract

Alzheimer’s disease (AD) is a progressive irreversible neurodegenerative disorder that represents a major global public health concern. Traditionally, AD is diagnosed using cerebrospinal fluid biomarker analysis or brain imaging modalities. Recently, less burdensome, more widely available blood biomarker (BBM) assays for amyloid-beta (Aβ42/40) and phosphorylated-tau concentrations have been found to accurately identify the presence/absence of brain amyloid plaques and tau tangles and have helped to streamline AD diagnosis. However, few BBMs have been rigorously analytically validated. Herein, we report the analytical validation of a novel liquid chromatography–tandem mass spectrometry (LC-MS/MS) multiplex method for quantifying plasma phosphorylated-tau217 (p-tau217) and non-phosphorylated-tau217 (np-tau217) peptide concentrations. We combined the p-tau217/np-tau217 concentrations ratio (%p-tau217) and the previously validated LC-MS/MS multiplex assay for plasma Aβ42/40 into a new multianalyte assay with algorithmic analysis (MAAA; PrecivityAD2™ test) that identifies brain amyloid status based on brain amyloid positron emission tomography. We found (a) the %p-tau217 assay is precise, accurate, sensitive, and linear over a wide analytical measurement range, and free from carryover and interference; (b) the pre-analytical specimen collection, processing, storage, and shipping conditions that maintain plasma tau peptide stability; and (c) using the measured analytical imprecision for plasma Aβ42/40 and p-tau217/np-tau217 levels in a worst-case scenario model, the PrecivityAD2 test algorithm for amyloid pathology classification changed for only 3.5% of participants from brain amyloid positive to negative, or from negative to positive. The plasma sample preparation and LC-MS/MS methods underlying the PrecivityAD2 test are suitable for use in the clinical laboratory and valid for the test’s intended purpose: to aid in the diagnostic evaluation of individuals aged 55 and older with signs or symptoms of mild cognitive impairment or dementia.

## 1. Introduction

Alzheimer’s disease (AD) is a serious neurodegenerative disorder that leads to progressive cognitive decline and dementia. It is irreversibly debilitating, destroys memory and thinking skills, and impairs individuals’ ability to carry out tasks of daily living. AD is the most common cause of dementia, accounting for 60% to 80% of dementia cases [1]. An estimated 7 million Americans and 40 to 50 million people globally are living with AD in 2024, and more than 90% of these individuals are ≥65 years old [1]. Driven by population aging, AD dementia prevalence is expected to increase significantly in the next three decades, placing major demands on caregivers and healthcare services and financial pressure on payers. Unless the disease can be effectively treated or prevented, healthcare systems around the world are in danger of becoming overwhelmed by the future costs of caring for people with AD and related dementias.

Despite the public health burden, mild cognitive impairment and dementia due to AD are underdiagnosed and misdiagnosed by clinicians and are underreported by patients and families. Only one-half of individuals who meet the criteria for dementia are clinically diagnosed [2]. When a diagnosis does occur, it is typically at a relatively late stage in the disease process when cognitive impairment and disability are prominent and less amenable to treatment. In addition to depriving symptomatic individuals of timely care, under- or misdiagnosis deprives policymakers of accurate AD incidence and prevalence data necessary to plan the infrastructure of care, services, and interventions. Thus, a timely and accurate AD diagnosis is reasonable and necessary to provide symptomatic individuals with optimal medical and nonmedical care and policymakers with quality data to guide decision making.

In response to the need for accurate and accessible BBMs to aid in the diagnosis of AD, the PrecivityAD^®^ blood test was commercially introduced in 2020 as the first analytically and clinically validated blood test for clinical care [3,4,5,6,7,8]. This test relies on immunoprecipitation liquid chromatography–tandem mass spectrometry (LC-MS/MS) to precisely quantify plasma amyloid beta (Aβ) isoforms Aβ42 and Aβ40 concentrations, and to determine the apolipoprotein E proteotype. Combined with patient age into a proprietary algorithm, an amyloid probability score (APS) ranging from 0 to 100 is calculated to identify the likelihood that a patient has brain amyloid pathology (a hallmark of AD) as defined by amyloid imaging [4,6,7,8].

Recent evidence suggests that the plasma concentrations of tau phosphorylated at threonine-217 (p-tau217) and the concentrations ratio of p-tau217 to non-phosphorylated-tau217 (np-tau217) expressed as a percentage (%p-tau217) further improves the diagnostic performance of BBMs (Aβ42/40 and others) for detecting brain amyloid and, potentially, brain neurofibrillary tau tangles [9,10,11]. In addition to our LC-MS/MS multiplex platform for quantifying plasma Aβ42/40, we have developed and optimized a second novel immunoprecipitation LC-MS/MS multiplex method that quantifies plasma p-tau217 and np-tau217 levels to calculate plasma %p-tau217. We have combined the plasma Aβ42/40 and %p-tau217 values quantified using two separate LC-MS/MS multiplex assays into a second-generation multianalyte assay with algorithmic analysis (MAAA) known as the PrecivityAD2™ test. This test produces an APS2 value on a 0–100 scale to identify brain amyloid status with a high degree of accuracy among both high-risk asymptomatic individuals [9,12] and patients with cognitive symptoms attending primary and secondary care centers for clinical evaluation [13]. These data, along with other published data [14,15,16] support the test’s intended use in individuals aged 55 years or older with signs or symptoms of cognitive impairment undergoing evaluation for AD [14,15,16].

Herein, we present the analytical validation of the p-tau217 and np-tau217 LC-MS/MS assay. We also evaluated how variations in the pre-analytical blood sample collection, processing, storage, and shipping procedures affect plasma p-tau217 and np-tau217 stability. Finally, we modeled the effects of the analytical variability associated with our Aβ42/40 and %p-tau217 measures on the APS2 algorithm among the 583 individuals previously analyzed as part of the test’s clinical validation in the intended use patient population [14].

## 2. Materials and Methods

### 2.1. Blood Sample Collection

Venous whole blood was collected into K_2_ EDTA-containing vacutainers^®^ and within 30–40 min of collection centrifuged for 15 min at 500–1500× *g* at room temperature. Within 240 min of phlebotomy, plasma was aliquoted into labeled 2.0 mL screw-cap polypropylene cryovials (Sarstedt Inc., Newton, NC, USA; part# 72.694.700) and frozen on dry ice or at −70 °C to −80 °C until analysis.

### 2.2. P-tau217 and np-tau217 Quantification

On the day of use, frozen calibrators, quality control (QC) samples, plasma test samples, trypsin endopeptidase (Millipore Sigma, St. Louis, MO, USA), and stable labeled ^15^N,^13^C p-tau internal standard (IS) peptides (Biosynth, Gardner, MA, USA) solutions were thawed. Immunoprecipitation buffers and IS peptides (in concentrations that mimicked the mid-range of expected p-tau217 and np-tau217 concentrations) were added to each 2 mL well on a 96-well low protein binding plate (Thermo Fisher Scientific, Waltham, MA, USA). In total, 900 µL of plasma test sample, QC plasma, or calibrators were added to assigned positions on the 96-well plate. A slurry that contained a proprietary monoclonal antibody conjugated to tosyl magnetic beads (Invitrogen, Waltham, MA, USA) was added to each well for tau protein immunocapture. After 60 min, the magnetic beads bound to tau were removed from the matrix and washed with PBS to reduce non-specifically bound contaminants prior to enzymatic digestion.

After washing, the tau-bound magnetic beads were placed in a temperature-controlled trypsin endopeptidase buffer solution, where plasma and IS tau protein species were digested into specific peptides. After 120 min at 37 °C, the digestion reaction was quenched with formic acid (FA). Tau digested samples were further purified using reverse-phase solid-phase extraction (SPE), washed with 0.1% FA to remove contaminants, and the tau peptides were eluted using 10% acetonitrile (ACN)/0.1% FA. The 96-well collection plate was dried under vacuum before samples were reconstituted in 16 µL of 0.5% ACN/0.1% FA.

The 96-well collection plate containing the resolubilized tau peptides was placed in a temperature-controlled autosampler within the LC system (Waters Acquity UPLC M-Class), and 4.5 µL of the reconstituted samples were injected onto the analytical LC column (Waters Corp., Milford, MA, USA; C18HSS), where they were separated, identified, and quantified using liquid chromatography–electrospray ionization mass spectrometry (Waters Acquity UPLC M-Class liquid chromatography unit interfaced to a Thermo Scientific Fusion Lumos Tribrid mass spectrometer). Phospho- and np-tau peptide concentrations were calculated by the summation of peak areas from monitored product ions that result after fragmentation of their respective precursor ions. Precursor ions derived from exogenous IS and endogenous peptides that corresponded to phosphorylated and non-phosphorylated tau peptides containing amino acids 212–221. After summation, the total peak areas for the endogenous tau peptides were divided by the total peak areas for the corresponding exogenously added IS peptides to obtain peak area ratios (PAR). The PAR of the endogenous peptides to the PAR of their respective IS peptides were determined in plasma samples, and the concentration of each endogenous peptide was calculated using PAR from calibration curves. These data were assembled and assessed using TraceFinder 4.1 General Quan software (Thermo Fisher Scientific).

### 2.3. P-tau217 and np-tau217 Calibration and Quality Control

Each validation run included five calibrators for each measurand, plus the matrix blank (Table 1), six QC samples, and plasma test samples.

Calibrators were prepared by spiking 2% recombinant human serum albumin (rHSA) with known amounts of synthetic tau peptides that contained p-tau217 and np-tau217 (Biosynth). The concentrations of the synthetic tau peptide stock solutions used to prepare the calibrators were value-assigned by USP-traceable amino acid analysis.

The six QC samples covered the anticipated high, medium, and low plasma concentration ranges for p-tau217 and np-tau217 peptides. QC multi-rules were applied: a run was rejected when one or more QC concentrations fell outside of 3 standard deviations (SD), or two or more QC sample concentrations exceeded 2 SDs, or if the range of two or more QCs exceeded 4 SDs (1-3S, 2-2S, R-4S rule). Total allowable error (TEa) for np-tau217 was defined as 30% or 15 pg/mL, whichever was greater, while TEa for p-tau217 was set at the greater of 30% or 0.3 pg/mL.

### 2.4. Precision

A nested multifactor analysis of variance (ANOVA) design was used to evaluate within-run precision (repeatability) and total (within-laboratory) precision [17]. The study used a 10 × 2 × 2 design where test samples and calibrators were analyzed over 10 days, with two runs per day, two replicates of each test sample per run, using a single lot of reagents and a single LC-MS/MS system. A minimum of two independent lots of calibrators were used with recalibration after five days. Four plasma test samples were analyzed per measurand to cover most of the analytical measurement range (AMR):

(1) Native, non-pooled, non-spiked human plasma sample, near the detection limits of both p-tau217 and np-tau217.
(2) Non-pooled human plasma sample, spiked with concentrations of p-tau217 and np-tau217 between the anticipated limit of detection (LoD) and midpoint of the calibration curve.
(3) Native, non-pooled, non-spiked human plasma sample, near the midpoint of the calibration curve.
(4) Non-pooled human plasma sample, spiked with concentrations of p-tau217 and np-tau217 between midpoint of the curve and the calibrators with the highest p-tau217 and np-tau217 concentrations.

The rationale for including two modified plasma samples was to create surrogate samples with high p-tau217 and np-tau217 concentrations, which are difficult to obtain in large quantities from commercial suppliers. The two modified plasma samples were spiked with known amounts of both p-tau217 and np-tau217 USP-traceable recombinant protein standards. Data were analyzed by multifactor ANOVA. Acceptable total (within-laboratory) imprecision was ½ TEa (7.5 pg/mL or ≤15% for np-tau217 and 0.15 pg/mL or ≤15% for p-tau217).

### 2.5. Analytical Measurement Range (AMR)

P-tau217 and np-tau217 concentrations were equally distributed over five levels. Samples were analyzed using one reagent lot and one LC-MS/MS system. The low concentration pool (Level 1) consisted of 2% rHSA containing p-tau217 and np-tau217 below the LoD of the assay. For both measurands, the high concentration pool was created by spiking 2% rHSA with synthetic tau peptide standards that contained p-tau217 and np-tau217 to achieve a concentration slightly higher than that of the highest concentrations previously observed in human plasma samples (Level 5). The volume of synthetic tau peptide standard solution spiked into the plasma pool did not exceed 5% of the total specimen volume to minimize matrix differences. Five test samples consisted of the low concentration sample (Level 1), the high concentration sample (Level 5), and three admixtures that incorporated different amounts of the Level 1 and Level 5 samples (Table 2).

Each level was prepared and analyzed in duplicate in randomized order. Polynomial regression analysis was performed at the first, second, and third order as specified [18]. Ideally, the measured values fit a first-order regression model without statistically significant nonlinear coefficients, but deviation from linearity less than or equal to ^1^/_3_ TEa (≤10% or 0.1 pg/mL for p-tau217 and ≤10% or 5 pg/mL for np-tau217) was considered acceptable.

### 2.6. Trueness/Accuracy

There are no certified reference materials for p-tau217 and np-tau217 measurands in a human plasma or serum matrix. Therefore, trueness for plasma tau peptide measurements was assessed by recovery experiments from native plasma samples spiked with known amounts of commercially available tau peptide standards, either phosphorylated at threonine-217 or not phosphorylated at threonine-217 (Biosynth). Tau peptide standards were value-assigned by a USP-traceable amino acid analysis, as described previously [4]. Tau peptide standards were spiked into three plasma test samples with low endogenous levels of p-tau217 and np-tau217 to achieve final concentrations of 2, 4, and 8 pg/mL or 50, 150, and 250 pg/mL for p-tau217 and np-tau217, respectively. Unspiked and spiked plasma samples were analyzed in triplicate. This analytical test was performed on two different LC-MS/MS systems each with a different reagent lot. Recovery of the spiked materials was calculated by subtracting the average concentration of the endogenous analyte (unspiked plasma sample) from the average concentration of the spiked plasma samples. Percent recovery was calculated by taking the ratio of the spiked concentration to the expected concentration. Recovery was considered acceptable if p-tau217 and np-tau217 recovery was less than ½ TEa: between 0.15 pg/mL and 7.5 pg/mL, respectively, or 85% to 115%.

### 2.7. Carryover Assessment

Two samples were tested for injection-to-injection carryover on the LC system: one sample concentration approximately five-fold higher than the highest concentration observed in human samples and one sample concentration near the LoD of the assay. The high-concentration sample was created by spiking 2% rHSA with synthetic tau peptide standards to achieve a concentration of 125 pg/mL p-tau217 and 1130 pg/mL np-tau217. The low-concentration sample was created by spiking 2% rHSA with synthetic tau peptide standards to achieve a final concentration near the LoD (0.5 pg/mL for p-tau217 and 7.9 pg/mL for np-tau217). Testing was performed in a single analytical run using the following sequence: five replicate analyses of the low-concentration sample (protected low pool), followed by one analysis of the high-concentration sample, followed by one analysis of the low-concentration sample (unprotected low). This “high, low” analytical sequence was repeated five times. Percentage carryover was calculated as (concentration of unprotected low sample—average concentration of protected low sample analyses)/(average concentration of high concentration sample analyses) × 100%.

Carryover < 2% was considered clinically insignificant and acceptable.

### 2.8. Sensitivity: Limit of Blank (LoB), Limit of Detection (LoD), and Limit of Quantitation (LoQ)

The LoB is the highest expected value in a series of sample replicates that contain no analyte. This study used a single “blank” sample (2% rHSA) analyzed on two LC-MS/MS systems over three days with two runs per day using two different reagent lots, one lot for each LC-MS/MS system. The blank sample was analyzed three times per run for 36 total measurements. The CLSI equation was used to calculate LoB [19]:

LoB = mean_Blank_ + (Cβ × SD_Blank_); (1)

where Cβ is a correction for the biased estimate of the population standard deviation (SD) due to sample size:

(2) $$C\beta =\frac{1.645}{1-(1/(4\times df))}$$

df = degrees of freedom; df = n − 1.

n = number of sample measurements.

The LoD study followed a similar design. A 3 × 2 × 3 design was used in which three low-concentration samples were analyzed on two LC-MS/MS systems over three days with three test sample replicates per run using two reagent lots (one reagent lot per LC-MS/MS system). Test samples were prepared using native human plasma or 2% rHSA spiked with synthetic tau peptide standards to concentrations near the expected LoD. The CLSI equation used to calculate LoD is [19]:

LoD = LoB + (Cβ × SD_LoD_), (3)

where Cβ is defined as above;

df = n − k − 1, k = # samples, n = # replicates per sample.

The LoQ was defined as the lowest actual concentration at which the measurands are reliably detected with an imprecision no more than 20%. If the %CV for the samples used in the LoD experiment is ≤20%, then the LoQ is equal to the LoD.

### 2.9. Extended Measuring Interval (Clinically Reportable Range)

The clinically reportable range (CRR) is the range of analyte values that a method can measure, allowing for specimen dilution, concentration, or another pretreatment used to extend the direct AMR [20]. Rarely, a patient’s plasma p-tau217 or np-tau217 concentration will exceed the defined AMR and the plasma will need to be diluted to bring that patient’s p-tau217 or np-tau217 concentrations within the AMR. This experiment was conducted in one run using one reagent lot and one LC-MS/MS system. Due to the scarcity of native human plasma samples with very high endogenous p-tau217 and np-tau217 concentrations, 2% rHSA was spiked with synthetic tau peptide standards at concentrations approximately 3 times higher than the highest-concentration calibrator, to create three samples containing both p-tau217 and np-tau217. Briefly, four dilutions were prepared and analyzed. Using the notation s/t, where s is the relative volume of the spiked plasma and t is the total volume after the diluent was added, the s/t ratios tested in this experiment were 1/4, 1/8, and 1/16, each measured in duplicate. The duplicate analyses of each dilution point were averaged and divided against the target value. Dilution was considered acceptable if recovery for each dilution was within ½ TEa (np-tau217 recovery within 7.5 pg/mL or 85% to 115%; p-tau217 recovery within 0.15 pg/mL or 85% to 115%).

### 2.10. Specificity and Interference

Studies were performed to determine if specific metabolites and common drugs, when added to the sample matrix, interfered with p-tau217 and np-tau217 quantitation. Potential interferents (Table 3), at recommended test concentrations [21], were spiked into two native plasma samples that had high and low endogenous p-tau217 and np-tau217 concentrations as per CLSI protocol [22]. Each of the four concentration combinations were analyzed in triplicate: low p-tau217 and np-tau217 concentrations with high interferent concentrations, and high p-tau217 and np-tau217 concentrations with high interferent concentrations.

Stock solutions of concentrated interferents, roughly 20-fold higher than the test concentration, or as concentrated as possible, were prepared using materials purchased from Sigma-Aldrich (St. Louis, MO, USA) or Sun Diagnostics (New Gloucester, ME, USA). The test samples were created by spiking the plasma pools with a specific amount of interferent stock solution to yield desired final concentrations of respective interferents. Control native human plasma samples were created by adding the appropriate solvent in a volume equal to the interferent spike volume. Using one LC-MS/MS system, five replicate test samples that contained an interferent and the corresponding control samples were analyzed on one day using a single reagent lot. The difference between the mean measurand concentration values for the test and control samples was calculated and then divided by the mean measurand value for the control samples and multiplied by 100 to calculate percent bias. Interference was considered clinically insignificant if deviation between the spiked and non-spiked sample did not exceed ½ TEa (difference ≤ 7.5 pg/mL or 15% for np-tau217; difference ≤ 0.15 pg/mL or 15% for p-tau217).

### 2.11. Sample Collection, Processing, and Analyte Stability Prior to Analysis

The effect of various blood sample collection and processing conditions on the stability of plasma p-tau217 and np-tau217 concentrations was assessed in cognitively impaired and age-matched cognitively normal participants (*N* = 40).

This study protocol and an informed consent document were reviewed and approved by an AAHRPP-accredited Institutional Review Board (Advarra). The protocol was described to each participant or legal representative, and both provided verbal and written informed consent prior to participation. Participants were enrolled and specimens collected and processed at a single clinical research center in Orlando, FL. Here, the phlebotomist collected 110 mL of blood into eleven × 10 mL K_2_ EDTA tubes. The “gold standard” sample collection procedure requires blood collection into a 10 mL K_2_ EDTA tube, centrifugation within 30 min of blood collection to obtain plasma, immediate aliquoting into polypropylene microfuge tubes, and freezing on dry ice or at −80 °C. Frozen samples are shipped on dry ice or cold packs to the laboratory using an overnight express courier.

We evaluated p-tau217 and np-tau217 tolerance to potential deviations in specimen collection, processing, storage, and shipping conditions that differed from the “gold standard” procedure (Table 4). Portions of each participant’s 110 mL blood sample were processed, stored, and shipped using the gold standard procedure and multiple variations of these pre-analytical conditions that might affect analyte stability. The plasma p-tau217 and np-tau217 concentrations quantified in specimens that were not handled according to the gold standard procedure were compared to the analyte concentrations quantified in the specimens handled according to the gold standard procedure.

Briefly, blood collection tubes were centrifuged to separate plasma at different timepoints after phlebotomy, aliquoted into microfuge tubes at different timepoints after centrifugation, and refrigerated at 4–8 °C or frozen by placing in dry ice after various timepoints. For each condition, each participant’s plasma was transferred to a 15 mL polypropylene tube for mixing (to assure a homogeneous sample) before aliquoting into 0.5 mL polypropylene microfuge tubes. After specimen processing was completed, the collected blood or plasma microfuge tubes were shipped for analysis on the day of collection on cold pack (“wet ice”) or dry ice. Laboratory personnel were blinded to all participants’ demographics, collection time points, and sample processing conditions while preparing and analyzing samples. Plasma samples were analyzed for p-tau217 and np-tau217 as described above.

For each collection/processing/storage/shipping condition, the average difference (bias) in p-tau217 and np-tau217 concentrations between each condition vs. those for the gold standard condition was calculated across all 40 participants. These concentration values were compared (paired *t*-test), and if the values were not significantly different (*p* > 0.05), or the average bias from the gold standard collection condition (#1) was less than or equal to 7.5% (¼ TEa), then the collection and shipping conditions were considered acceptable.

### 2.12. Plasma Sample Freeze–Thaw Study

Six participants with plasma collected using the gold standard conditions (plasma centrifuged within 30 min, frozen, and shipped on dry ice) were chosen for this study. Twelve 1 mL aliquots were split into groups and placed into four separate freezer boxes, stored at −80 °C, and subjected to four freeze–thaw cycles. On each of three days, a subset of samples was thawed, mixed by inversion, and uncapped to mimic actual use. Thawed sample tubes were then recapped and returned to −80 °C freezer. After all freeze–thaw cycles were completed, all samples were removed from −80 °C storage, thawed, and analyzed as described above. This isochronous design allowed us to compare the effects of each freeze–thaw cycle on measurand concentrations quantified in the same analytical run (one 96-well plate) to minimize potential inter-day variability. Acceptance criteria were defined as a mean difference ≤¼ TEa, or 7.5%, or two-tailed paired *t*-test *p*-value > 0.05.

### 2.13. Modeling Variability on PrecivityAD2 Algorithm

The PrecivityAD2 algorithm (APS2) is a logistic regression model that combines Aβ42/40 and %p-tau217 measures to output the likelihood of the presence or absence of brain amyloid in an individual. To assess the effect of the analytical imprecision associated with plasma Aβ42, Aβ40, p-tau217, and np-tau217 measures on the diagnostic classification of an individual, we conducted a sensitivity analysis by modeling plasma analyte levels obtained from 583 previously studied individuals with MCI or dementia [14]. The mean total %CV measured during analytical validation for each of the four analytes (7.85% for p-tau217, 9.03% for np-tau217, 4.24% for Aβ40, and 4.92% for Aβ42 [4]) was applied in a normally distributed fashion to the actual data from the 583 individuals. One hundred simulated sample results were generated per participant, and the Aβ42/40 and %p-tau217 values were used to calculate APS2 values for the simulated samples. The two Aβ (Aβ42 and 40) and p-tau217 (p-tau217 and np-tau217) values were independently randomly generated, allowing for the measures to vary in opposite directions. To assess the effect of analytical variation, we calculated the percent of samples in the modeled data set where the APS2 status changed amyloid positive values to amyloid negative and amyloid negative values to positive near the established APS2 cutoff value (47.5) when compared to the original APS2 value calculated for each participant.

## 3. Results

### 3.1. Sensitivity

The LoB was 2 pg/mL and 0.3 pg/mL for np-tau217 and p-tau217, respectively. The LoD was 7 pg/mL and 1.3 pg/mL for np-tau217 and p-tau217, respectively. The LoQ for both p-tau217 and np-tau217 was equal to the LoD.

### 3.2. Precision

The results for the four samples of each analyte, analyzed over 5 days, with two runs per day, and two replicates per run, are presented in Table 5. Total imprecision (within-lab) for np-tau217 varied from 5.4% to 9.9%. Total imprecision (within-lab) for p-tau217 varied from 7.3% to 9.7%. Within-day imprecision (repeatability) for np-tau217 varied from 6.0% to 8.8%, while within-day (repeatability) for p-tau217 varied from 7.3% to 12.0%.

### 3.3. Accuracy

Trueness for plasma np-tau217 and p-tau217 was assessed using recovery experiments. Table 6 outlines acceptable recovery for low (96–108%), medium (102–113%), and high concentration (100–114%) spike and recovery experiments for both p-tau217 and np-tau217.

### 3.4. Interference

None of the potential interferents tested interfered with p-tau217 or np-tau217 quantitation up to the concentrations tested (Table 3).

### 3.5. Analytical Measurement Range, Clinically Reportable Range (CRR), and Carryover

**Figure 1:**
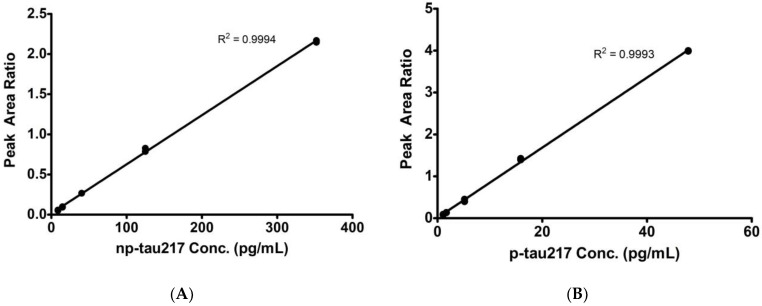
Calibration curves for (**A**) np-tau217 and (**B**) p-tau217.

**Figure 2:**
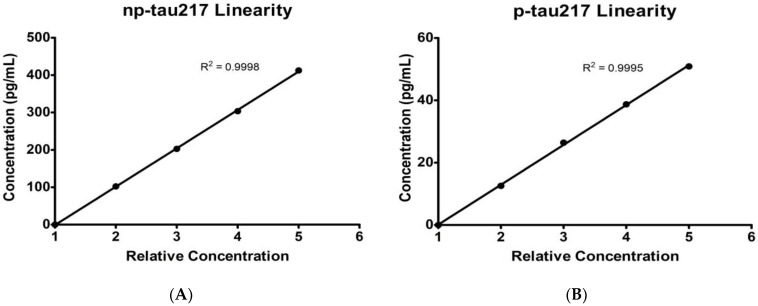
Linearity over Levels 1–5 for (**A**) np-tau217 and (**B**) p-tau217.

Typical calibration curves are shown in Figure 1. P-tau217 concentrations were linear from 0.1 to 81 pg/mL, and np-tau217 concentrations were linear from 3 to 280 pg/mL (Figure 2).

For test samples with exceptionally high p-tau217 and np-tau217 concentrations, three dilutions were tested: 1/4, 1/8, and 1/16, each measured in duplicate. Up to a 16-fold dilution was found to be acceptable for both analytes (Table 7).

Therefore, based on the LoD, lower limit of linearity, dilution studies, and current concentrations of the highest calibrator, the CRR is defined for np-tau217 as 7–4480 pg/mL and for p-tau217 as 0.5–1296 pg/mL.

Carryover was ≤2% and not considered clinically relevant (Table 8).

### 3.6. Sample Collection, Processing, and Stability

Blood sample collection, processing, and shipping conditions, as summarized in Table 4, demonstrated that in comparison to the established gold standard collection, processing, storage, and shipping conditions: (1) whole blood samples shipped with cold packs are acceptable for reliable analysis if received and centrifuged within 24 h of collection; (2) EDTA plasma is acceptable for reliable analysis if shipped with cold packs and received within 48 h after blood collection; (3) whole blood must be centrifuged to EDTA plasma within 180 min after blood draw for reliable analysis; and (4) EDTA plasma specimens must be frozen within 48 h of phlebotomy in order to provide reliable p-tau217 and np-tau217 concentrations (Table 9).

Up to four freeze–thaw cycles did not significantly affect p-tau217 or np-tau217 concentrations.

### 3.7. Modeling the Effect of Analytical Variability on the Diagnostic Result

**Figure 3:**
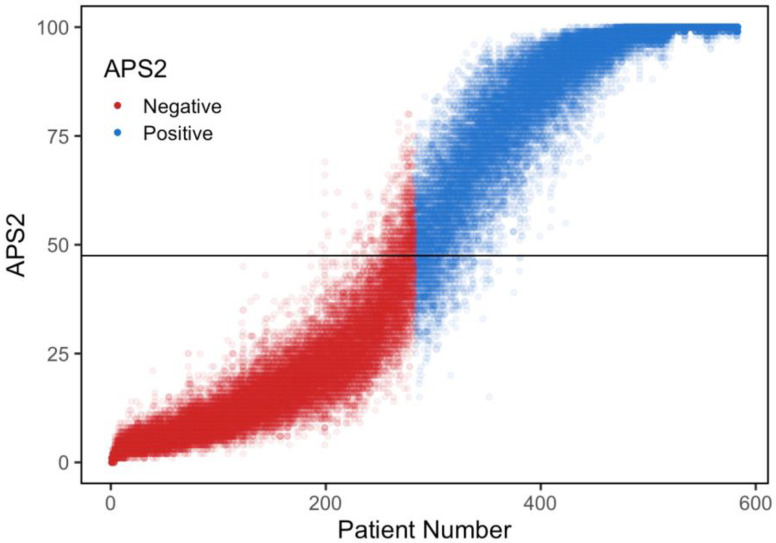
Modeled APS2 values for each of the 583 participants enrolled in the clinical validation of plasma Aβ42/40 and %p-tau217. The measured analytical imprecision was applied to each analyte for each of the 583 individuals who were ranked by their originally calculated APS2 value from 0 (absence of brain amyloid pathology) to 100 (presence of brain amyloid pathology). Each dot represents one of the 100 APS2 values modeled for each patient, and the color of the dot represents the original APS2 status based on each patient’s actual biomarker measurements. Red points (brain amyloid negative) above the clinically validated cut point (horizontal line at 47.5) and blue points below the cut point represent 3.5% of the simulated data points where the diagnostic classification for that individual changed from brain amyloid negative to positive or from brain amyloid positive to negative due to the analytical imprecision for the four analytes.

Using the measured analytical variability (within-lab mean total imprecision; %CV) for the four analytes used to calculate the APS2, we assessed the worst-case effect of analytical imprecision on the test’s interpretation (Figure 3), by allowing the four test components to vary completely independently. Plasma Aβ isoform analytical variability was previously published [4]. APS2 is a whole number between 0 and 100. Among MCI individuals for whom the PrecivityAD2 test is indicated, APS2 values > 47.5 represent a positive result and a high likelihood of brain amyloid presence; APS2 values < 47.5 represent a negative result and a low likelihood of brain amyloid presence. The algorithm and the 47.5 optimal cut point were previously validated against amyloid PET imaging [14].

For each of the simulated model sample results, the original APS2 status in terms of being above (amyloid positive) or below (amyloid negative) 47.5 was compared to the APS2 recalculated from the modeled analyte values (Figure 3). From this simulation, we calculated that 96.5% of the samples in this cohort had no change in their APS2 interpretation when modeling the worst-case scenario for analytical imprecision of the four analytes. Figure 3 shows that the amyloid pathology classification (amyloid positive vs. negative) for samples with APS2 values closer to the floor or ceiling of the scale (0 or 100, respectively) are largely unaffected by the measured analytical imprecision.

## 4. Discussion

The analytical validation metrics reported here demonstrate that plasma sample collection, processing, storage, shipping, preparation, and analytical procedures developed to quantify p-tau217 and np-tau217 in K_2_ EDTA plasma are precise, linear, specific, and free from carryover and interferences. In combination with other analytically and clinically validated plasma biomarkers quantified using separate sample preparation and LC-MS/MS multiplex procedures (Aβ42, Aβ40), plasma %p-tau217 substantially adds to the robust diagnostic performance of the PrecivityAD2 blood test to identify the likelihood of brain amyloid pathology [9,13,14,15]. Recent evidence suggests that quantifying plasma p-tau species can also aid in the detection of brain neurofibrillary tau tangles [9,10,11,23,24,25,26,27,28,29,30], another biochemical hallmark for AD.

High-performance BBMs like the PrecivityAD2 test have the potential to simplify and streamline testing for the presence of brain amyloid pathology among individuals with signs or symptoms of cognitive impairment undergoing evaluation for AD. Access to these analytically and clinically validated blood tests is expected to save healthcare dollars, reduce patient burden and time to diagnosis, identify eligibility status for clinical trials, predict future cognitive decline, and facilitate access to approved therapies for cognitively impaired individuals [3,31,32,33,34].

Multiple lines of evidence support the notion that combining plasma p-tau217 and Aβ42/40 measures enhances the identification of brain amyloid pathology, particularly at the earliest stages [35,36,37,38,39,40]. Some evidence suggests that quantifying other plasma p-tau species besides p-tau217 (e.g., p-tau181, p-tau205, p-tau212, p-tau231, p-tau235) might also identify cerebral amyloid pathology, but not quite as well as p-tau217 or %p-tau217 [41,42,43,44,45,46]. In agreement with others, our preliminary experiments indicated that %p-tau217 is considerably more robust, reliable, and concordant with brain amyloid pathology than p-tau181 or %p-tau181 [9,14]. Our analytical and clinical validations of plasma %p-tau217, Aβ42/40 assays, and the APS2 algorithm value demonstrate that these biomarkers identify brain amyloid pathology at least as well, if not better, than several other plasma biomarker assays for p-tau217 and other p-tau species [10,12,15,32,41,47,48,49,50].

It is curious that plasma p-tau217 is a biomarker for brain amyloid pathology. However, several plasma p-tau217 and p-tau217/np-tau217 assays have consistently performed well by detecting AD pathology in patients with mild cognitive impairment and dementia, and recently in cognitively unimpaired adults [9,10,11,12,13,14,15,16]. Taken together and based on the A/T/N classification scheme for BBMs of brain amyloid pathology [51], the above evidence and recent findings [11,12] support the working hypothesis that plasma Aβ42/40 is an early biomarker for brain amyloid dysmetabolism and amyloid plaque formation that precedes or signals neuronal death, phospho-tau tangle formation, and phospho-tau release into the plasma and CSF [52,53]. It is also important to note that several tau PET imaging studies have correlated the presence of cerebral tau tangles with plasma p-tau measures [10,30,54]. This reinforces the rationale for combining validated plasma Aβ42/40 and %p-tau217 measures into a prediction model that best identifies A+/T− and A+/T+ individuals as they progress through the hypothesized amyloid cascade of pathological changes that define AD [9,55,56].

The study limitations include the lack of a direct head-to-head comparison of the described sample preparation and quantification for p-tau217 and np-tau217 measurement with other analytical platforms that quantify p-tau217. However, many other platforms do not quantify np-tau217, so a direct comparison may not be valid. Regardless, both a prototype LC-MS/MS assay for p-tau217/np-tau217 and the current plasma %p-tau217 had superior clinical performance when compared to several other p-tau217 and total-tau immunoassays [15,41]. The possibility exists that plasma p-tau217/np-tau217 values normalize for inter-individual differences in np-tau217 (a surrogate for total non-phosphorylated tau) and adjusts for inter-individual differences in plasma p-tau217 handling due to renal clearance or other comorbid conditions that commonly occur with advanced age [57,58,59,60].

## 5. Conclusions

We provide experimental evidence that confirms the analytical validity of a novel LC-MS/MS assay that quantifies plasma p-tau217 and np-tau217 concentrations, which when combined with our previously validated plasma Aβ42/40 measures [3,4,6,7,8], can accurately identify brain amyloid pathology. We define pre-analytical conditions under which plasma p-tau217 and np-tau217 concentrations are stable and unaffected by select time and temperature variations that might occur during collection, processing, preparation, shipping, and freeze/thaw cycles. Finally, we demonstrate that the measured analytical imprecision associated with plasma preparation, immunoprecipitation, and LC-MS/MS quantitation of Aβ42, Aβ40, p-tau217, and np-tau217 does not dramatically affect the ability of these biomarkers to identify brain amyloid pathology when they are used to calculate a score (APS2) that accurately reflects the likelihood of brain amyloid pathology presence or absence. Further study will clarify the ability of this biomarker combination and additional biomarkers for identifying brain tau pathology and other neurodegenerative disorders.

## Figures and Tables

**Table 1:** p-tau217 and np-tau217 calibrator concentrations.

Calibrator	p-tau217 (pg/mL)	np-tau217 (pg/mL)
A	0.0	0.0
B	0.09	1.15
C	12.6	102
D	26.4	203
E	38.7	304
F	50.9	412

The range of calibrators used to quantify each measurand were as follows: p-tau217; Calibrators A and C–F, np-tau217; Calibrators A–E.

**Table 2:** Admixture proportional mixing strategy for five equally spaced test sample concentrations.

Level	% Low Pool	% High Pool
1	100	0
2	75	25
3	50	50
4	25	75
5	0	100

**Table 3:** List of potential interfering substances and maximum interferent concentrations tested.

Potential Interfering Substance	Maximum Test Concentration
**Endogenous**	
Human proteins (albumin and gamma-globulins; 50:50 mix)	12.5 g/dL
Bilirubin (unconjugated)	40 mg/dL
Hemolysate (Hemoglobin)	1000 mg/dL
Triglyceride-rich lipoproteins	1000 mg/dL
Rheumatoid factor (RF)	1000 U/mL
Human anti-mouse antibody (HAMA)	1:200 titer
**Exogenous**	
Acetaminophen	15.6 mg/dL
Aspirin (acetylsalicylic acid)	3.0 mg/dL
Atorvastatin	0.075 mg/dL
Citalopram	0.54 mg/dL
Clonazepam	0.030 mg/dL
Donepezil	100 ng/mL
Ibuprofen	21.9 mg/dL
Memantine	0.0117 mg/dL
Risperidone	0.0114 mg/dL
Valproic Acid	31.8 mg/dL

**Table 4:** Blood collection, processing, storage, and shipping conditions tested.

Condition #	Description
1	Blood centrifuged w/in 30 min, plasma shipped on dry ice (gold standard condition).
2	Whole blood shipped on cold pack, processed within 24 h of collection.
3	Whole blood shipped on cold pack, processed within 48 h of collection.
4	Whole blood shipped on cold pack, processed within 49 h of collection.
5	Blood centrifuged w/in 30 min, plasma shipped on cold pack, frozen within 24 h of collection.
6	Blood centrifuged w/in 30 min, plasma shipped on cold pack, frozen within 48 h of collection.
7	Blood centrifuged w/in 30 min, plasma shipped on cold pack, frozen within 49 h of collection.
8	Blood centrifuged w/in 120 min, plasma shipped on cold pack, frozen within 24 h of collection.
9	Blood centrifuged w/in 120 min, plasma shipped on cold pack, frozen within 48 h of collection.
10	Blood centrifuged w/in 120 min, plasma shipped on cold pack, frozen within 49 h of collection.
11	Blood centrifuged w/in 180 min, plasma shipped on cold pack, frozen within 24 h of collection.
12	Blood centrifuged w/in 180 min, plasma shipped on cold pack, frozen within 48 h of collection.
13	Blood centrifuged w/in 180 min, plasma shipped on cold pack, frozen within 49 h of collection.

**Table 5:** Plasma np-tau217 and p-tau217 precision.

**np-tau217**	**Mean pg/mL**	**Within-Lab (Total)**		**Within-Day (Repeatability)**	
**Level**		**SD, pg/mL**	**CV**	**SD, pg/mL**	**CV**
1	20.0	1.4	7.1%	1.2	6.0%
2	52.3	4.7	9.0%	3.4	7.5%
3	95.3	5.2	5.4%	2.2	6.1%
4	303.1	29.8	9.9%	12.0	8.8%
**p-tau217**	**Mean pg/mL**	**Within-Lab (Total)**		**Within-Day (Repeatability)**	
**Level**		**SD, pg/mL**	**CV**	**SD, pg/mL**	**CV**
1	1.53	0.15	9.7%	0.05	8.5%
2	5.39	0.39	7.3%	0.01	7.3%
3	21.66	2.14	9.7%	1.36	12.0%
4	37.32	3.49	9.4%	1.25	9.5%

%CV = (SD/Mean) × 100.

**Table 6:** np-tau217 and p-tau217 recovery experiments.

**np-tau217**		**Instr 1/Lot 1**		**Instr 1/Lot 2**		**Instr 2/Lot 1**		**Instr 2/Lot 2**	
	**Added, pg/mL**	**Obs, pg/mL**	**Recovery %**	**Obs, pg/mL**	**Recovery %**	**Obs, pg/mL**	**Recovery %**	**Obs, pg/mL**	**Recovery %**
Base Pool	0	38.77		73.13		35.12		69.81	
Low	50	92.86	105%	133.6	108%	88.12	104%	126.0	105%
Med	150	212.8	113%	237.4	106%	204.3	110%	223.1	102%
High	250	311.2	108%	351.0	109%	307.2	108%	320.4	100%
**p-tau217**		**Instr 1/Lot 1**		**Instr 1/Lot 2**		**Instr 2/Lot 1**		**Instr 2/Lot 2**	
	**Added, pg/mL**	**Obs, pg/mL**	**Recovery %**	**Obs, pg/mL**	**Recovery %**	**Obs, pg/mL**	**Recovery %**	**Obs, pg/mL**	**Recovery %**
Base Pool	0	1.31		1.75		1.31		1.71	
Low	2	3.23	98%	3.62	96%	3.21	97%	3.83	103%
Med	4	5.98	113%	6.01	104%	6.01	113%	6.36	112%
High	8	10.16	109%	9.91	102%	10.64	114%	10.26	106%

Instr = Instrument #; Obs = Observed concentration.

**Table 7:** np-tau217 and p-tau217 dilution and recovery experiments.

**np-tau217**	**Expected**	**Measured**	
**Dilution**	**Conc. (pg/mL)**	**Conc. (pg/mL)**	**Recovery %**
Undiluted	1561	1561	
1:4	390	382	98%
1:8	195	202	104%
1:16	98	97	99%
**p-tau217**	**Expected**	**Measured**	
**Dilution**	**Conc. (pg/mL)**	**Conc. (pg/mL)**	**Recovery %**
Undiluted	50	50.3	
1:4	12.5	12.3	98%
1:8	6.3	6.5	103%
1:16	3.1	3.4	110%

**Table 8:** np-tau217 and p-tau217 carryover study.

	Protected		Unprotected	
	Low Pool	High Pool	Low Pool	%Carryover
Mean np-tau217, pg/mL	7.9	1129.9	8.7	0.07%
Mean p-tau217, pg/mL	0.48	124.62	0.51	0.00%

**Table 9:** Bias attributable to variations in specimen collection, processing, storage, and shipping conditions.

	np-tau217			p-tau217			%p-tau217		
Condition #	Mean pg/mL	Mean Bias	*p*-Value	Mean pg/mL	Mean Bias	*p*-Value	Mean pg/mL	Mean Bias	*p*-Value
1	88.4			0.53			1.10		
2	91.6	3.2	0.2349	0.65	0.12	0.4197	1.27	0.16	0.4269
3	94.8	6.4	**0.0327**	0.33	−0.20	0.5594	0.59	−0.51	0.0839
4	94.8	6.4	**0.0221**	0.52	−0.01	0.2713	0.89	−0.22	**0.0463**
5	89.4	1.0	0.7230	0.60	0.06	0.4029	1.22	0.12	0.6361
6	90.2	1.8	0.5073	0.44	−0.09	0.7229	0.83	−0.27	0.8121
7	89.3	0.9	0.8049	0.58	0.05	0.8565	1.11	0.00	0.9136
8	87.8	−0.6	0.8224	0.97	0.44	0.8463	1.82	0.72	0.7782
9	87.6	−0.8	0.6713	0.28	−0.25	0.5980	0.44	−0.66	0.6210
10	87.9	−0.5	0.8638	0.61	0.08	0.1302	1.24	0.14	0.1190
11	92.8	4.4	0.3091	0.86	0.33	0.6298	1.87	0.77	0.3363
12	90.4	2.0	0.6214	0.42	−0.11	0.5196	0.96	−0.14	0.8988
13	89.3	0.9	0.7992	0.32	−0.21	0.6893	0.67	−0.44	0.8815

Condition numbers are described in Table 4. Bolded *p*-Values indicate that the Mean value (pg/mL) for that Condition# is statistically different from the Mean value for Condition #1.

## Data Availability

The datasets used and/or analyzed during the current study are available from the corresponding author upon reasonable request.

## Abbreviations

AD;	Alzheimer’s disease
ACN;	acetonitrile
Aβ40;	amyloid beta-40
Aβ42;	amyloid beta-42
APS2;	amyloid probability score 2
AMR;	analytical measurement range
A/T/N;	Amyloid/Tau/Neurodegeneration classification scheme
BBM;	blood biomarker
CRR;	clinically reportable range
CSF;	cerebrospinal fluid
CV;	coefficient of variation
FA;	formic acid
F/T;	freeze/thaw
IP;	immunoprecipitation
IS;	internal standard
K_2_ EDTA;	dipotassium ethylene diamine tetraacetic acid
LoB;	limit of blank
LoD;	limit of detection
LoQ;	limit of quantitation
LC;	liquid chromatography
LC-MS/MS;	liquid chromatography-tandem mass spectrometry
MAAA;	multianalyte assay with algorithmic analysis
MCI;	mild cognitive impairment
PAR;	peak area ratio
PET;	positron emission tomography
np-tau217;	non-phosphorylated tau217 peptide
p-tau;	phosphorylated tau peptide
p-tau217;	phosphorylated tau217 peptide
%p-tau217;	p-tau217/np-tau217 concentrations ratio
QC;	quality control
rHSA;	recombinant human serum albumin
SD;	standard deviation
SST;	system suitability test
SPE;	solid phase extraction
TEa;	total allowable error
USP;	United States Pharmacopeia

## References

[1] Alzheimer’s Association (2024). 2024 Alzheimer’s Disease Facts and Figures. Alzheimer’s Dement..

[2] Amjad H., Roth D.L., Sheehan O.C., Lyketsos C.G., Wolff J.L., Samus Q.M. (2018). Underdiagnosis of Dementia: An Observational Study of Patterns in Diagnosis and Awareness in US Older Adults. J. Gen. Intern. Med..

[3] Canestaro W.J., Bateman R.J., Holtzman D.M., Monane M., Braunstein J.B. (2024). Use of a Blood Biomarker Test Improves Economic Utility in the Evaluation of Older Patients Presenting with Cognitive Impairment. Popul. Health Manag..

[4] Kirmess K.M., Meyer M.R., Holubasch M.S., Knapik S.S., Hu Y., Jackson E.N., Harpstrite S.E., Verghese P.B., West T., Fogelman I. (2021). The PrecivityAD^TM^ Test: Accurate and Reliable LC-MS/MS Assays for Quantifying Plasma Amyloid Beta 40 and 42 and Apolipoprotein E Proteotype for the Assessment of Brain Amyloidosis. Clin. Chim. Acta.

[5] Monane M., Johnson K.G., Snider B.J., Turner R.S., Drake J.D., Maraganore D.M., Bicksel J.L., Jacobs D.H., Ortega J.L., Henderson J. (2023). A Blood Biomarker Test for Brain Amyloid Impacts the Clinical Evaluation of Cognitive Impairment. Ann. Clin. Transl. Neurol..

[6] West T., Kirmess K.M., Meyer M.R., Holubasch M.S., Knapik S.S., Hu Y., Contois J.H., Jackson E.N., Harpstrite S.E., Bateman R.J. (2021). A Blood-Based Diagnostic Test Incorporating Plasma Aβ42/40 Ratio, ApoE Proteotype, and Age Accurately Identifies Brain Amyloid Status: Findings from a Multi Cohort Validity Analysis. Mol. Neurodegener..

[7] Fogelman I., West T., Braunstein J.B., Verghese P.B., Kirmess K.M., Meyer M.R., Contois J.H., Shobin E., Ferber K.L., Gagnon J. (2023). Independent Study Demonstrates Amyloid Probability Score Accurately Indicates Amyloid Pathology. Ann. Clin. Transl. Neurol..

[8] Hu Y., Kirmess K.M., Meyer M.R., Rabinovici G.D., Gatsonis C., Siegel B.A., Whitmer R.A., Apgar C., Hanna L., Kanekiyo M. (2022). Assessment of a Plasma Amyloid Probability Score to Estimate Amyloid Positron Emission Tomography Findings Among Adults with Cognitive Impairment. JAMA Netw. Open.

[9] Rissman R.A., Langford O., Raman R., Donohue M.C., Abdel-Latif S., Meyer M.R., Wente-Roth T., Kirmess K.M., Ngolab J., Winston C.N. (2024). Plasma Aβ42/Aβ40 and Phospho-tau217 Concentration Ratios Increase the Accuracy of Amyloid PET Classification in Preclinical Alzheimer’s Disease. Alzheimer’s Dement..

[10] Mattsson-Carlgren N., Janelidze S., Bateman R.J., Smith R., Stomrud E., Serrano G.E., Reiman E.M., Palmqvist S., Dage J.L., Beach T.G. (2021). Soluble P-Tau217 Reflects Amyloid and Tau Pathology and Mediates the Association of Amyloid with Tau. EMBO Mol. Med..

[11] Barthélemy N.R., Salvadó G., Schindler S.E., He Y., Janelidze S., Collij L.E., Saef B., Henson R.L., Chen C.D., Gordon B.A. (2024). Highly Accurate Blood Test for Alzheimer’s Disease Is Similar or Superior to Clinical Cerebrospinal Fluid Tests. Nat. Med..

[12] Janelidze S., Barthélemy N.R., Salvadó G., Schindler S.E., Palmqvist S., Mattsson-Carlgren N., Braunstein J.B., Ovod V., Bollinger J.G., He Y. (2024). Plasma Phosphorylated Tau 217 and Aβ42/40 to Predict Early Brain Aβ Accumulation in People Without Cognitive Impairment. JAMA Neurol..

[13] Palmqvist S., Tideman P., Mattsson-Carlgren N., Schindler S.E., Smith R., Ossenkoppele R., Calling S., West T., Monane M., Verghese P.B. (2024). Blood Biomarkers to Detect Alzheimer Disease in Primary Care and Secondary Care. JAMA.

[14] Meyer M.R., Kirmess K.M., Eastwood S., Wente-Roth T.L., Irvin F., Holubasch M.S., Venkatesh V., Fogelman I., Monane M., Hanna L. (2024). Clinical Validation of the PrecivityAD2 Blood Test: A Mass Spectrometry-based Test with Algorithm Combining %p-tau217 and Aβ42/40 Ratio to Identify Presence of Brain Amyloid. Alzheimer’s Dement..

[15] Schindler S.E., Petersen K.K., Saef B., Tosun D., Shaw L.M., Zetterberg H., Dage J.L., Ferber K., Triana-Baltzer G., Du-Cuny L. (2024). Head-to-Head Comparison of Leading Blood Tests for Alzheimer’s Disease Pathology. medRxiv.

[16] Warmenhoven N., Salvadó G., Janelidze S., Mattsson-Carlgren N., Bali D., Dolado A.O., Kolb H., Triana-Baltzer G., Barthélemy N.R., Schindler S.E. (2024). A Comprehensive Head-to-Head Comparison of Key Plasma Phosphorylated Tau 217 Biomarker Tests. medRxiv.

[17] Clinical and Laboratory Standards Institute (CLSI) (2014). EP05-A3. Evaluation of Precision of Quantitative Measurement Procedures.

[18] Clinical and Laboratory Standards Institute (CLSI) (2003). EP06-A. Evaluation of the Linearity of Quantitative Measurement Procedures: A Statistical Approach.

[19] Clinical and Laboratory Standards Institute (CLSI) (2012). EP17. Evaluation of Detection Capability for Clinical Laboratory Measurement Procedures.

[20] Clinical and Laboratory Standards Institute (CLSI) (2018). EP34. Establishing and Verifying an Extended Measuring Interval Through Specimen Dilution and Spiking.

[21] Clinical and Laboratory Standards Institute (CLSI) (2018). EP37. Supplemental Tables for Interference Testing in Clinical Chemistry.

[22] Clinical and Laboratory Standards Institute (CLSI) (2018). EP07. Interference Testing in Clinical Chemistry.

[23] Groot C., Smith R., Stomrud E., Binette A.P., Leuzy A., Wuestefeld A., Wisse L.E.M., Palmqvist S., Mattsson-Carlgren N., Janelidze S. (2023). Phospho-Tau with Subthreshold Tau-PET Predicts Increased Tau Accumulation Rates in Amyloid-Positive Individuals. Brain.

[24] Coomans E.M., Verberk I.M.W., Ossenkoppele R., Verfaillie S.C.J., Visser D., Gouda M., Tuncel H., Wolters E.E., Timmers T., Windhorst A.D. (2023). A Head-to-Head Comparison Between Plasma PTau181 and Tau PET Along the Alzheimer’s Disease Continuum. J. Nucl. Med..

[25] Jack C.R., Wiste H.J., Algeciras-Schimnich A., Figdore D.J., Schwarz C.G., Lowe V.J., Ramanan V.K., Vemuri P., Mielke M.M., Knopman D.S. (2023). Predicting Amyloid PET and Tau PET Stages with Plasma Biomarkers. Brain.

[26] Mielke M.M., Hagen C.E., Xu J., Chai X., Vemuri P., Lowe V.J., Airey D.C., Knopman D.S., Roberts R.O., Machulda M.M. (2018). Plasma Phospho-tau181 Increases with Alzheimer’s Disease Clinical Severity and Is Associated with Tau- and Amyloid-positron Emission Tomography. Alzheimer’s Dement..

[27] Mundada N.S., Rojas J.C., Vandevrede L., Thijssen E.H., Iaccarino L., Okoye O.C., Shankar R., Soleimani-Meigooni D.N., Lago A.L., Miller B.L. (2023). Head-to-Head Comparison between Plasma p-Tau217 and Flortaucipir-PET in Amyloid-Positive Patients with Cognitive Impairment. Alzheimers Res. Ther..

[28] Woo M.S., Tissot C., Lantero-Rodriguez J., Snellman A., Therriault J., Rahmouni N., Macedo A.C., Servaes S., Wang Y., Arias J.F. (2023). Plasma PTau-217 and N-terminal Tau (NTA) Enhance Sensitivity to Identify Tau PET Positivity in Amyloid-β Positive Individuals. Alzheimer’s Dement..

[29] Ossenkoppele R., Reimand J., Smith R., Leuzy A., Strandberg O., Palmqvist S., Stomrud E., Zetterberg H., Scheltens P., Dage J.L. (2021). Tau PET Correlates with Different Alzheimer’s Disease-related Features Compared to CSF and Plasma P-tau Biomarkers. EMBO Mol. Med..

[30] Mielke M.M., Frank R.D., Dage J.L., Jeromin A., Ashton N.J., Blennow K., Karikari T.K., Vanmechelen E., Zetterberg H., Algeciras-Schimnich A. (2021). Comparison of Plasma Phosphorylated Tau Species with Amyloid and Tau Positron Emission Tomography, Neurodegeneration, Vascular Pathology, and Cognitive Outcomes. JAMA Neurol..

[31] Hampel H., Hu Y., Cummings J., Mattke S., Iwatsubo T., Nakamura A., Vellas B., O’Bryant S., Shaw L.M., Cho M. (2023). Blood-Based Biomarkers for Alzheimer’s Disease: Current State and Future Use in a Transformed Global Healthcare Landscape. Neuron.

[32] Schindler S.E., Li Y., Li M., Despotis A., Park E., Vittert L., Hamilton B.H., Womack K.B., Saef B., Holtzman D.M. (2023). Using Alzheimer’s Disease Blood Tests to Accelerate Clinical Trial Enrollment. Alzheimer’s Dement..

[33] Cullen N.C., Leuzy A., Janelidze S., Palmqvist S., Svenningsson A.L., Stomrud E., Dage J.L., Mattsson-Carlgren N., Hansson O. (2021). Plasma Biomarkers of Alzheimer’s Disease Improve Prediction of Cognitive Decline in Cognitively Unimpaired Elderly Populations. Nat. Commun..

[34] Zetterberg H., Blennow K. (2021). Moving Fluid Biomarkers for Alzheimer’s Disease from Research Tools to Routine Clinical Diagnostics. Mol. Neurodegener..

[35] Jack C.R., Wiste H.J., Algeciras-Schimnich A., Weigand S.D., Figdore D.J., Lowe V.J., Vemuri P., Graff-Radford J., Ramanan V.K., Knopman D.S. (2024). Comparison of Plasma Biomarkers and Amyloid PET for Predicting Memory Decline in Cognitively Unimpaired Individuals. Alzheimer’s Dement..

[36] Li D., Mielke M.M. (2019). An Update on Blood-Based Markers of Alzheimer’s Disease Using the SiMoA Platform. Neurol. Ther..

[37] Benedet A.L., Brum W.S., Hansson O., Karikari T.K., Zimmer E.R., Zetterberg H., Blennow K., Ashton N.J. (2022). The Accuracy and Robustness of Plasma Biomarker Models for Amyloid PET Positivity. Alzheimers Res. Ther..

[38] Pichet Binette A., Janelidze S., Cullen N., Dage J.L., Bateman R.J., Zetterberg H., Blennow K., Stomrud E., Mattsson-Carlgren N., Hansson O. (2023). Confounding Factors of Alzheimer’s Disease Plasma Biomarkers and Their Impact on Clinical Performance. Alzheimer’s Dement..

[39] Janelidze S., Palmqvist S., Leuzy A., Stomrud E., Verberk I.M.W., Zetterberg H., Ashton N.J., Pesini P., Sarasa L., Allué J.A. (2022). Detecting Amyloid Positivity in Early Alzheimer’s Disease Using Combinations of Plasma Aβ42/Aβ40 and P-tau. Alzheimer’s Dement..

[40] Palmqvist S., Stomrud E., Cullen N., Janelidze S., Manuilova E., Jethwa A., Bittner T., Eichenlaub U., Suridjan I., Kollmorgen G. (2023). An Accurate Fully Automated Panel of Plasma Biomarkers for Alzheimer’s Disease. Alzheimer’s Dement..

[41] Janelidze S., Bali D., Ashton N.J., Barthélemy N.R., Vanbrabant J., Stoops E., Vanmechelen E., He Y., Dolado A.O., Triana-Baltzer G. (2023). Head-to-Head Comparison of 10 Plasma Phospho-Tau Assays in Prodromal Alzheimer’s Disease. Brain.

[42] Karikari T.K., Ashton N.J., Brinkmalm G., Brum W.S., Benedet A.L., Montoliu-Gaya L., Lantero-Rodriguez J., Pascoal T.A., Suárez-Calvet M., Rosa-Neto P. (2022). Blood Phospho-Tau in Alzheimer Disease: Analysis, Interpretation, and Clinical Utility. Nat. Rev. Neurol..

[43] O’Connor A., Karikari T.K., Poole T., Ashton N.J., Lantero Rodriguez J., Khatun A., Swift I., Heslegrave A.J., Abel E., Chung E. (2020). Plasma Phospho-Tau181 in Presymptomatic and Symptomatic Familial Alzheimer’s Disease: A Longitudinal Cohort Study. Mol. Psychiatry.

[44] Palmqvist S., Tideman P., Cullen N., Zetterberg H., Blennow K., Dage J.L., Stomrud E., Janelidze S., Mattsson-Carlgren N., Hansson O. (2021). Prediction of Future Alzheimer’s Disease Dementia Using Plasma Phospho-Tau Combined with Other Accessible Measures. Nat. Med..

[45] Tsantzali I., Foska A., Sideri E., Routsi E., Tsomaka E., Kitsos D.K., Zompola C., Bonakis A., Giannopoulos S., Voumvourakis K.I. (2022). Plasma Phospho-Tau-181 as a Diagnostic Aid in Alzheimer’s Disease. Biomedicines.

[46] Neddens J., Temmel M., Flunkert S., Kerschbaumer B., Hoeller C., Loeffler T., Niederkofler V., Daum G., Attems J., Hutter-Paier B. (2018). Phosphorylation of Different Tau Sites during Progression of Alzheimer’s Disease. Acta Neuropathol. Commun..

[47] Ashton N.J., Pascoal T.A., Karikari T.K., Benedet A.L., Lantero-Rodriguez J., Brinkmalm G., Snellman A., Schöll M., Troakes C., Hye A. (2021). Plasma P-Tau231: A New Biomarker for Incipient Alzheimer’s Disease Pathology. Acta Neuropathol..

[48] Lantero-Rodriguez J., Salvadó G., Snellman A., Montoliu-Gaya L., Brum W.S., Benedet A.L., Mattsson-Carlgren N., Tideman P., Janelidze S., Palmqvist S. (2024). Plasma N-Terminal Containing Tau Fragments (NTA-Tau): A Biomarker of Tau Deposition in Alzheimer’s Disease. Mol. Neurodegener..

[49] Montoliu-Gaya L., Benedet A.L., Tissot C., Vrillon A., Ashton N.J., Brum W.S., Lantero-Rodriguez J., Stevenson J., Nilsson J., Sauer M. (2023). Mass Spectrometric Simultaneous Quantification of Tau Species in Plasma Shows Differential Associations with Amyloid and Tau Pathologies. Nat. Aging.

[50] Milà-Alomà M., Ashton N.J., Shekari M., Salvadó G., Ortiz-Romero P., Montoliu-Gaya L., Benedet A.L., Karikari T.K., Lantero-Rodriguez J., Vanmechelen E. (2022). Plasma P-Tau231 and p-Tau217 as State Markers of Amyloid-β Pathology in Preclinical Alzheimer’s Disease. Nat. Med..

[51] Jack C.R., Bennett D.A., Blennow K., Carrillo M.C., Feldman H.H., Frisoni G.B., Hampel H., Jagust W.J., Johnson K.A., Knopman D.S. (2016). A/T/N: An Unbiased Descriptive Classification Scheme for Alzheimer Disease Biomarkers. Neurology.

[52] Cummings J., Zhou Y., Lee G., Zhong K., Fonseca J., Cheng F. (2023). Alzheimer’s Disease Drug Development Pipeline: 2023. Alzheimer’s Dement. Transl. Res. Clin. Interv..

[53] Aisen P.S., Cummings J., Jack C.R., Morris J.C., Sperling R., Frölich L., Jones R.W., Dowsett S.A., Matthews B.R., Raskin J. (2017). On the Path to 2025: Understanding the Alzheimer’s Disease Continuum. Alzheimers Res. Ther..

[54] Janelidze S., Berron D., Smith R., Strandberg O., Proctor N.K., Dage J.L., Stomrud E., Palmqvist S., Mattsson-Carlgren N., Hansson O. (2021). Associations of Plasma Phospho-Tau217 Levels with Tau Positron Emission Tomography in Early Alzheimer Disease. JAMA Neurol..

[55] Park J.-C., Han S.-H., Yi D., Byun M.S., Lee J.H., Jang S., Ko K., Jeon S.Y., Lee Y.-S., Kim Y.K. (2019). Plasma Tau/Amyloid-Β1–42 Ratio Predicts Brain Tau Deposition and Neurodegeneration in Alzheimer’s Disease. Brain.

[56] Mattsson-Carlgren N., Janelidze S., Palmqvist S., Cullen N., Svenningsson A.L., Strandberg O., Mengel D., Walsh D.M., Stomrud E., Dage J.L. (2021). Longitudinal Plasma P-Tau217 Is Increased in Early Stages of Alzheimer’s Disease. Brain.

[57] Gronewold J., Klafki H.-W., Baldelli E., Kaltwasser B., Seidel U.K., Todica O., Volsek M., Haußmann U., Wiltfang J., Kribben A. (2016). Factors Responsible for Plasma β-Amyloid Accumulation in Chronic Kidney Disease. Mol. Neurobiol..

[58] Stocker H., Beyer L., Trares K., Perna L., Rujescu D., Holleczek B., Beyreuther K., Gerwert K., Schöttker B., Brenner H. (2023). Association of Kidney Function with Development of Alzheimer Disease and Other Dementias and Dementia-Related Blood Biomarkers. JAMA Netw. Open.

[59] Janelidze S., Barthélemy N.R., He Y., Bateman R.J., Hansson O. (2023). Mitigating the Associations of Kidney Dysfunction with Blood Biomarkers of Alzheimer Disease by Using Phosphorylated Tau to Total Tau Ratios. JAMA Neurol..

[60] Arvanitakis Z., Lucas J.A., Younkin L.H., Younkin S.G., Graff-Radford N.R. (2002). Serum Creatinine Levels Correlate with Plasma Amyloid β Protein. Alzheimer Dis. Assoc. Disord..

